# Epidemic hemorrhagic fever complicated with late pregnancy: A case report

**DOI:** 10.1097/MD.0000000000008137

**Published:** 2017-10-27

**Authors:** Xiao-li Liu, Dong Hao, Xiao-Zhi Wang, Tao Wang, Feng Lu, Weili Liu, Bingjie Lv, Chang-Jun Lv

**Affiliations:** a Department of Shandong University School of Medicine, Jinan; b Department of Respiratory Disease (Taishan Scholar Position), Binzhou Medical University Hospital, Binzhou, China.

**Keywords:** epidemic hemorrhagic fever, hantavirus, hemorrhagic fever with renal syndrome, pregnancy

## Abstract

**Rationale:**

Hantaviruses cause two forms of diseases in humans, namely hemorrhagic fever with renal syndrome (HFRS) and hantavirus pulmonary syndrome. Hantavirus infections can occur in pregnant women, and could influence the maternal and fetal outcomes, although this is a rare finding, even in endemic areas.

**Patient concerns:**

In this report, we describe anunusual case involving a pregnant woman with HFRS who was in a state of shock.

**Diagnoses:**

Hemorrhagic fever with renal syndrome and septic shock.

**Interventions:**

Timely termination of pregnancyalong with correction of the shock is very important to curb the inflammation and reduce organ damage.

**Outcomes:**

Although HFRS in pregnancy could pose a serious threat to the lives of the mother and the child. Our patient was successfully treated.

**Lessons:**

Early and accurate diagnosis, anti-shock treatment, and timely termination of pregnancyare the key aspects of therapy for HFRS with late pregnancy.

## 1 Introduction

Hemorrhagic fever with renal syndrome, also called epidemic hemorrhagic fever (EHF), is a disease caused by species of hantaviruses. A common route of infection is the consumption of food contaminated by mouse urine or stool. HFRS is a Class B infectious disease, with a mortality rate of 3% to 10%. Although HFRS is epidemic in China, pregnancy along with HFRS is rare in China as well as in other countries. HFRS in pregnancy shows clinical manifestations similar to those of a variety of pregnancy-related complications. Failure of timely and accurate diagnosis of HFRS in pregnancy could pose a serious threat to the lives of the mother and the child. Here, we describe a case of HFRS complicating pregnancy in our hospital that was successfully treated.

## 2 Case report

Because the study didn’t involve the clinical trials, we have not applied for approval by the Ethics Committee. But we received the consent of the patient and her husband to make it known to public.

A 24-year-old previously healthy woman (gravida 0, para 0) was admitted to our hospital at 39 weeks of gestation on November 2, 2016 owing to fever, chills, and cough since 3 days. About 3 days before admission, the patient had suffered from fever of up to 39.4 °C, with chills. She began to cough, and experienced malaise, headache, and blurred vision. Blood examination conducted at a local hospital showed the following results: white blood cell (WBC), 6.1 × 10^9^/L (normal range, NR: 4–10 × 10^9^/L); red blood cell (RBC), 3.70 × 10^12^/L (NR: female, 3.5–5.0 × 10^12^/L); hemoglobin (HG), 114 g/L (NR: 110–140 g/L); platelets (PLT) 112 × 10^9^/L (NR: 100–300 × 10^9^/L). On examination after admission to our hospital, the blood pressure of the patient was 140/100 mmHg and her temperature was 38.8 °C. Arterial blood gas analysis at 29% inspired oxygen showed the following results: pH, 7.45; PaCO_2_, 14 mmHg; PaO_2_, 133 mmHg; sodium (Na^+^), 125 mmol/L (NR: 135–145 mmol/L); potassium, 3.0 mmol/L (NR: 3.5–5.5 mmol/L); lactic acid, 4.3 mmol/L (NR: <2 mmol/L); HCO_3_^–^, 9.7 mmol/L; SaO_2_, 99%. Laboratory investigations showed the following results: WBC, 22.93 × 10^9^/L; HG, 104 g/L; PLT, 14 × 10^9^/L; neutrophils, 85.74%; alanine aminotransferase, 51 U/L (NR: <40 U/L); aspartate aminotransferase, 177.9 U/L (NR: <50 U/L); albumin, 22.3 g/L (NR: 35–55 g/L); total bilirubin, 23.7 μmol/L; anion gap, 21.00 mmol/L (NR: 11–16 mmol/L); and lactate dehydrogenase, 1119.0 U/L (NR: 109–245 U/L). The blood sample of the patient tested positive for IgM antibody for EHF virus and negative for IgG antibody. The test was repeated 4 times. Her procalcitonin level was 1.14 ng/mL (NR: <0.25 ng/mL). Peripheral blood smear showed 3% abnormal lymphocytes and 1% broken red blood cells. Fetal ultrasound showed good fetal growth, which was consistent with the gestation dates.

Despite the administration of 1500 mL of physiologic saline for 4 hours, the condition of the patient deteriorated. Her blood pressure dropped to 80/50 mmHg, and the urine output was <30 mL/h. Her bulbar conjunctival congestion and edema were more obvious, with a flushed face and neck, and she was breathing rapidly. There were no other positive signs. Her WBC increased to 37.3 × 10^9^/L, but PLT was still 14 × 10^9^/L. Her urinary protein was +++. X-ray examination revealed the texture of both lungs. Hence, the patient was transferred to the intensive care unit (ICU) for further treatment. She received fluid resuscitation according to the sepsis bundle therapy. Subsequently, her central venous pressure (CVP) and lactic acid level were monitored to guide liquid management. She was administered sufficient albumin, electrolyte, and blood products such as plasma, cold precipitation, and fresh platelets for correction of coagulation abnormalities. In the first 20 hours, the patient accepted an intravenous intake of 6900 mL, and her urine output increased gradually after the first 10 hours, up to 200 mL/h. Her blood pressure increased to 100/60 mmHg and her lactic acid level normalized. Her CVP increased from 3 to 10 mmHg.

Fortunately, the oxygenation status of the patient was very good, with no lung injury. Subsequently, her biochemical enzyme levels decreased. However, at this moment, the patient already had a small amount of vaginal bleeding. She was administered 3 units of platelets to control the bleeding and protect the baby. Her PLT increased to 55 × 10^9^/L and quickly declined to 31 × 10^9^/L on Day 2. On Day 3, an obstetrician was contacted for consultation after the patient's shock had been corrected. After extensive discussion, a lower uterine segment cesarean section under combined spinal and epidural anesthesia was planned. Care was taken to prevent postpartum hemorrhage, and the obstetrician performed uterine artery ligation and uterine binding during the surgery. The fetus was successfully delivered, and his Apgar score was 10. The umbilical cord blood did not show epidemic hemorrhagic fever antibodies. After surgery, the patient was transferred back to the ICU; no obvious bleeding tendency was observed. After removal of tracheal intubation on Day 4, the patient seemed to breathe well, and her oxygenation index was about 280 mmHg. On Day 8, her PLT increased to 77 × 10^9^/L, and her blood sample was positive for epidemic hemorrhagic fever antibody IgG positive and weakly positive for IgM. Her urine sample was devoid of protein. She was transferred to the infection section. On Day 11, her PLT normalized to 170 × 10^9^/L, and she was discharged.

## 3 Discussion

Sepsis caused by viruses is not uncommon. The most obvious pathological changes in EHF are small blood vessels and renal lesions. The skin, mucous membranes, and the various systems and organs show a wide range of congestion, hemorrhage, and edema; in severe cases, necrosis formation is observed. The typical manifestations are fever, bleeding, and kidney damage as the 3 main signs; the condition usually shows a clinical process comprising 5 phases, namely fever, low pressure, less urine, more urine, and the recovery phase.^[1]^ However, in most cases, the clinical manifestation is not typical. As many families are unaware of infestation by rats, a history of contact with rats or mice is not a reliable basis for the diagnosis or exclusion of EHF. EHF-IgM can be used as the basis for the diagnosis of EHF.^[2]^ In the present case, although the contact history with mice was not assessed, our city, Binzhou, is a high-risk area for EHF; moreover, the patient often consumed restaurant food, which may have come in contact with rats or mice. The key point is that the patient had typical clinical symptoms of EHF and specific virus antibody (IgM). Her clinical manifestations were fever, headache, conjunctival congestion, and edema; thus, she experienced the 5 clinical phases of the condition. Laboratory tests showed an electrolyte imbalance, thrombocytopenia and coagulation dysfunction, liver dysfunction, and the presence of urinary proteins.

Pregnancy associated with EHF is rare, especially in developed countries. Pregnancy increases the organ burden of patients with EHF, worsens the condition, and increases the risk for adverse maternal outcomes. Studies have shown that maternal mortality rate is higher than that of non-pregnant women.^[3]^ Moreover, the EHF virus can infect the fetus through the placenta, posing a teratogenic risk to the fetus, and increasing the risk of stillbirth and other complications. Todorovic et al^[4]^ reported 2 cases of hantavirus infection in pregnant women whose babies were not infected. Figurnov et al^[5]^ reported a case of a pregnant woman infected with EHF at 31 weeks of gestation, whose children were found to be suffering from hydrocephalus, at a follow-up conducted 28 years later. However, Pettersson et al^[6]^ reported a case of hantavirus infection in Sweden, where the pregnant woman diagnosed with EHF at 3 weeks of gestation miscarried after 12 weeks. In 2 other cases, viral RNA was found in the breast milk of the lactating mother; however, the babies were healthy. In the current patient, EHF was detected in late pregnancy, that is, 39 weeks and 1 day, and no EHF antibody was found in the umbilical cord blood. We still need to track the growth and development of the infant.

Pregnancy with EHF is often complicated by conditions such as thrombocytopenia, coagulation disorders, elevated transaminases, and hypokalemia. This condition shares similarities with a variety of pregnancy-related complications such hemolysis, elevated liver enzymes and low platelets (HELLP) syndrome, acute fatty liver of pregnancy (AFLP),^[7]^ etc.; therefore, careful detection and distinction is important. HELLP syndrome is a common complication of pregnancy-induced hypertension. Its pathogenesis involves vasospasm, vascular endothelial damage, platelet activation, and hemolysis. The typical symptoms of HELLP syndrome are hemolysis, elevated liver enzymes, and thrombocytopenia, and its diagnosis depends on the clinical manifestations and laboratory examination. AFLP is a rare fatal disease in late pregnancy, characterized by acute onset and rapid changes in condition, and it resembles fulminant hepatitis. Its etiology is unknown, and coagulation dysfunction and thrombocytopenia are the typical manifestations. Early diagnosis and treatment, and early termination of pregnancy can reduce the mortality associated with AFLP. Patients with AFLP often have no fever or other signs of infection. Our patient had fever, no history of hypertension, and quickly entered the shock state; as she was positive for the specific viral antibody, the above diagnoses were not considered.

EHF has no specific treatment except for symptomatic treatment and supportive care. The prognosis of EHF in pregnancy depends on early and accurate diagnosis, and active comprehensive treatment, and whether the mother can safely go through the renal failure period. Timely termination of pregnancy along with correction of the shock is very important to curb the inflammation and reduce organ damage. Our experience in handling a large number of patients with septic shock helped in the successful treatment of this patient.

## References

[1] Gavrilovskaya IN Gorbunova EE Mackow NA Hantaviruses direct endothelial cell permeability by sensitizing cells to the vascular permeability factor VEGF, while angiopoietin 1 and sphingosine 1-phosphate inhibit hantavirus-directed permeability. J Virol 2008;82:5797–806. 18367532 10.1128/JVI.02397-07 PMC2395149

[2] Petraityte R Yang H Hunjan R Development and evaluation of serological assays for detection of Hantaanvirus-specific antibodies in human sera using yeast-expressed nucleocapsid protein. J Virol Methods 2008;148:89–95. 18077007 10.1016/j.jviromet.2007.10.019

[3] Klein SL Marks MA Li W Sex differences in the incidence and case fatality rates from hemorrhagic fever with renal syndrome in China, 2004–2008. Clin Infect Dis 2011;52:1414–21. 21628481 10.1093/cid/cir232 PMC3146012

[4] Todorovic Z Canovic P Gajovic O Hemorrhagic fever with renal syndrome during pregnancy: case report. Med Pregl 2010;63:280–4. 21053473 10.2298/mpns1004280t

[5] Figurnov VA Marunich NA Gavrilov AV Late consequences of hemorrhagic fever with renal syndrome in a woman who had it during pregnancy. Klin Med (Mosk) 2007;85:71–2. 17665611

[6] Pettersson L Boman J Juto P Outbreak of Puumala virus infection, Sweden. Emerg Infect Dis 2008;14:808–10. 18439368 10.3201/eid1405.071124 PMC2600231

[7] Mace G Feyeux C Mollard N Severe Seoul hantavirus infection in a pregnant woman, France, October 2012. Euro Surveill 2013;18:20464. 23647626

